# Sintered Brake Pads Failure in High-Energy Dissipation Braking Tests: A Post-Mortem Mechanical and Microstructural Analysis

**DOI:** 10.3390/ma16217006

**Published:** 2023-11-01

**Authors:** Alexandre Mege-Revil, Jessie Rapontchombo-Omanda, Itziar Serrano-Munoz, Anne-Lise Cristol, Vincent Magnier, Philippe Dufrenoy

**Affiliations:** 1 UMR 9013—LaMcube—Laboratoire de Mécanique, Multiphysique, Multiéchelle, Université de Lille, CNRS, Centrale Lille, F-59000 Lille, France; jessie.rapontchombo@hotmail.fr (J.R.-O.); itziar.serrano-munoz@bam.de (I.S.-M.); anne-lise.cristol@centralelille.fr (A.-L.C.); vincent.magnier@polytech-lille.fr (V.M.); philippe.dufrenoy@polytech-lille.fr (P.D.); 2 Bundesanstalt für Materialforschung und-Prüfung (BAM), Unter den Eichen 87, 12205 Berlin, Germany

**Keywords:** friction braking, sintering, metallic matrix pad, microstructure, SEM, compressive test, cracks, DIC, diffusion

## Abstract

The industrial sintering process used to produce metallic matrix pads has been altered to diminish the amount of copper used. Unfortunately, replacing a large part of the copper with iron seems to have reached a limit. In the high-energy, emergency-type rail braking used in this study, the materials are put to the very limit of their usage capacity, allowing us to observe the evolution of the microstructure and mechanical properties of sintered, metallic matrix pads. After the braking test, their compressive behaviour was assessed using digital image correlation (DIC), and their microstructure with scanning electron microscopy (SEM). The worn material has three flat layers with different microstructures and compressive behaviours. The bottom layer seems unmodified. Macroscopic and microscopic cracks run through the intermediate layer (2–15 mm depth). The top layer has stiffened thanks to resolidification of copper. The temperature reaches 1000 °C during the braking test, which also explains the carbon diffusion into iron that result in the weakening of iron –graphite interfaces in the pad. Finally, submicronic particles are detected at many open interfaces of the worn and compressed pad. Associated with the predominant role of graphite particles, this explains the weak compressive behaviour of the pads.

## 1. Introduction

Friction braking is a mechanical application that implies using strong, yet cheap materials. Whether for automotive or rail applications, the pad–disc system is confronted with the same requirements: dissipating kinetic energy as fast as possible, while maintaining stability in the contact with minimal occurrence of particle emissions, noise, and vibrations. The most usual choices as friction systems are cast iron or low-carbon alloyed steel discs versus composite pads. Wear and degradation mechanisms have been widely documented, highlighting the importance of the sub-surface in the generation of wear particles [1,2,3,4,5,6,7,8,9,10]. At the present time, most studies have focused on the mechanical behaviour of the disc, as it is the main source of wear particles, and needs replacing as soon as the stress level can induce the development of cracks. Either for cast-iron [11,12] or for steel discs [13,14,15,16], the very high temperature and very fast cooling that can be reached in some experimental conditions lead to the formation of bainite and martensite, and, thus, to more fragile mechanical behaviours.

Facing the disc, the pad is designed to meet very stringent specifications, typically leading to an excessively complex composition [8,10,17]. Most components are introduced in the hope of correcting friction parameters for greater efficiency in specific braking conditions [18,19,20], or simply to fill the matrix with a cheap, inert material. Depending on the nature of the matrix of the pad, two technologies have been developed: polymer matrices are usually favoured for moderate braking conditions, whereas metallic matrices are principally dedicated to high-energy applications [9,10,21]. Nonetheless, both types of matrices can be found in automotive and rail services applications. 

In the past years, following public and academic concern [22,23,24], health and safety regulations have evolved in many areas, including trying to ban copper from the composition [25,26]. The trend for metallic matrix pads has been to shift from copper to iron matrices. Unfortunately, iron matrices are tricky to sinter and prove to be less efficient [27], although some authors report stable, high coefficients of friction and low wear rates for relatively low amounts of iron [28]. As a consequence, most of the recently developed pads still show a fair amount of copper in combination with iron. 

This is curious engineering. Whatever the rest of the filling powders, sintering a mixture of iron and copper powders can be interpreted as an inadvertent, yet quite efficient attempt at favouring corrosion. First, the corrosion potential of iron is significantly lower than that of copper. Secondly, the sintering process implies creating direct, oxide-less contact between iron and copper, that is to say, a good electric connection. And thirdly, the same sintering process undoubtedly results in a fair volume of porosities, including open porosities. As the pads are quite obviously exposed to atmospheric humidity, including in some cases snow and salt, corrosion will happen.

Nonetheless, iron–copper matrix pads have passed the control tests and have been used on both cars and train braking systems for some years now. It is clear, from the few previous studies, that the mechanical properties of the break-pad strongly influence its durability [29,30]. In some cases, early degradation of the pad has been observed [31], with massive weight losses. Several studies [9,21] have investigated this phenomenon, concluding that the mechanical stress and the repartition of ceramics and graphite particles had a strong influence on the development of cracks several millimetres below the contact surface.

In a previous study [30], a finite element analysis was led to distinguish the mechanical behaviours of the components using an optimization algorithm. Coupled with 2D and 3D measurements, it provided information on the non-linear behaviour of our pads in a static compressive test and allowed us to identify the components that are involved in some mechanisms.

In this study, we aim at understanding the influence of the sintering process on the mechanical behaviour of sintered pads, in the context of a high-energy braking test series, by discussing the evolution of their microstructure. The compressive behaviour of a commercial pad produced using a simplified formulation is assessed in comparison with other materials missing one or several components. The brake-pad materials are examined before and after the braking tests via scanning electron microscopy (SEM) and subjected to compressive tests combined with digital image correlation (DIC). This procedure allows us to expose the metallurgical mechanisms that explain both the complex compressive behaviour and the development of major cracks in the pads during high-energy braking. 

## 2. Materials and Methods

### 2.1. Materials

A low carbon alloyed steel (15CrMoV5) disc was used for the braking test series. 

To facilitate our understanding of the roles of each component, a simplified version of a commercial metallic-matrix pad is used as reference material (RM). The selected components are detailed in Table 1 along with their sizes. G1 and G2 in the labels stand for Graphite 1 and Graphite 2 that are different in shapes, sizes, origin, and chemical purity. Graphite 1 is smaller (50 to 500 nm), less round, and comes from a recovery process (Graphite “4020”, Mersen Company, Gennevilliers, France) from machining operations of graphite parts, graphite scrap, and defective and broken graphite grains. Graphite 2 was provided by Imerys Graphite & Carbon Company (Paris, France, reference TIMREX KS 300-1250). As its name indicates, it ranges between 300 and 1250 µm in diameter. It is a synthetic material, highly pure and crystalline, owing to the very high temperature process.

Other pad materials were also sintered to be able to dissociate the role of the components. As given in Table 2, MMCsG1 and MMCsG2 (Metallic Matrix + Ceramics + Graphite) are similar to the RM (Reference Material), but with only one type of graphite instead of two, the missing amount of graphite being replaced by the same amount of the other kind, to reach 20% in mass. MM (Metallic Matrix) only contains the sintered metallic matrix with inert, undisclosed fillers. MMCs (Metallic Matrix + Ceramics) contains both ceramics added to it. These samples have the same relative proportions of each compound as the RM except for the missing compounds. 

The powders are mixed in an industrial device, then pressed for 5 s at room temperature under 285 MPa in the shape of the future pad. The final stage is the actual sintering process, which is set in a hydrogenated atmosphere to ensure reductive conditions. This is a 3-step process, starting with a one-hour step at 820 °C, a one-hour step at 870 °C and a final 8.5 MPa pressurised step at 955 °C. No pressure is applied during the cooling step, although a nitrogen flow is still brought in to avoid the presence of oxygen. 

### 2.2. Compression Tests

The five pad materials previously described (RM, MM, MMCs, MMCsG1, and MMCsG2) endured a compressive test along the z-axis, that is to say, the axis of the sintering process’ applied pressure. These tests were also performed on the RM pad material after a high-energy braking test that will be described in the next section.

An INSTRON-5500 (INSTRON, Elancourt, France) machine equipped with a load-sensor of 100 kN was used to compress 20 × 20 × 20 mm^3^ cubes for the case of the MMCsG1, MMCsG2, and RM samples. Due to their higher strength, the MM and MMCs samples were prepared in the shape of 5 × 5 × 10 mm^3^ prisms. Since graphite particles in the pad can reach several millimetres, the volume of the samples may seem slightly small to be truly representative of the heterogeneity of the pad. Nonetheless, several tests were performed on samples extracted in different areas of the pads, showing a great consistency in the results [32]. 

In order to evaluate their compressive behaviour, incremental, ten-loop loading steps were performed after preloading the sample at 1 MPa. In the case of MMCsG1, MMCsG2, and RM samples, a first ten-loop cycle was applied, reaching a moderate load (5 MPa). Then, a second ten-loop cycle was performed reaching a higher load (10 MPa), and the pattern was repeated to overcome the yield strength and progress into plastic deformation (15 and 20 MPa). For the MM and MMCs samples, for which the heterogeneity is less pronounced as there is no graphite, the stress values were higher than for the MMCsG1, MMCsG2, and RM samples to enable consistent measurements. The load steps during compressive tests were set at 100, 150, 200, and finally at 250 MPa. 

Three faces of the samples were carefully prepared to ensure a good measurement. The top and bottom surfaces were rectified to ensure good parallelism. The front lateral surface was ground down to P1200 grit paper before being sprayed in black, then airbrushed with contrasting white speckles to allow displacement measurement by Digital Image Correlation (DIC). A 2048 × 2048 pixels XIMEA camera (XIMEA GmbH, Münster, Germany) was used to record images of the 20 × 20 mm² speckle pattern at 2 Hz frequency. This technique is consistent with the displacement measured with the gauges [17]. In addition, it allows us to assess the heterogeneity of the displacement that is due to the heterogeneity of the mechanical properties of the components. Nonetheless, it is important to keep in mind that this technique gives us the displacement of the front surface, making us assume that the rest of the sample shows a similar, yet topographically distinct behaviour. This hypothesis seems reasonable to us, as the volume of the sample is sufficient in comparison with the maximum size of the grains to allow a sufficiently homogeneous distribution. Finally, the resolution of the camera is around 300 µm, which only allows interpretation on the chunky Graphite 2 particles, which are enclosed in a matrix where other components are indistinguishable. 

### 2.3. Braking Tests

A standard series of braking tests was applied to a RM pad. This program is detailed in [31] and follows guidelines from the Union Internationale des Chemins de fer (UIC). Three series of five brakings of increasing intensity were applied, following each time an initial series of five softer braking designed to reset the surface. In each series, the initial speed was increased from 80 to 250 km/h. The first series was set with a normal force of 5 kN, for a simulated mass of 5 t. The second series was set for a normal force of 36 kN for a simulated mass of 7 t. The last series was set for a normal force of 45 kN and a simulated mass of 9.5 t, reaching very severe conditions that represent a train emergency braking from 250 km/h to complete stop. The duration of brakings from the initial speed to complete stop was measured between 18 and 65 s. The temperature was monitored with rubbing thermocouples.

### 2.4. Microstructure Observations

For optical characterization, the samples were polished down to 1 µm diamond paste and chemically attacked for 5 s with a 4 %vol. solution of Nital. The observations were performed on a Nikon MA200 microscope (Tokyo, Japan). SEM measurements were also performed using a Hitachi 3600N microscope (Tokyo, Japan), equipped with an EDAX energy dispersive spectrometer. 

To gain knowledge on porosities, Synchrotron Computed Tomography (SCT) was carried out at the ID19 beamline of the European Synchrotron Radiation Facility (ESRF), Grenoble, France. Cylinders 5 mm in diameter and 18 mm high were cut out of the pad for this purpose, the height being the direction of compaction of the pad. The voxel size was 3.425 µm^3^ for a 110 keV scan.

## 3. Results

### 3.1. Microstructure and Defects of the Pads

**Figure 1:**
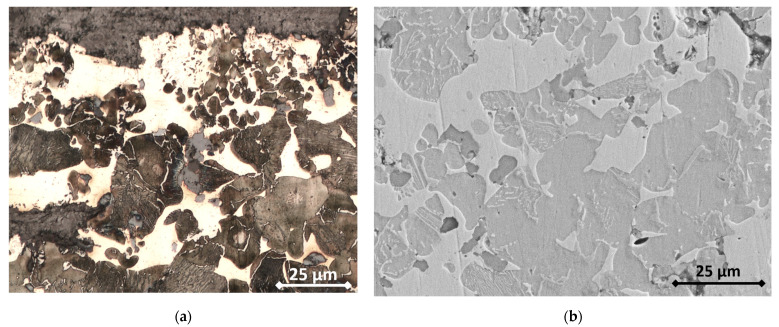
(**a**) Optical micrograph of the metallic matrix of the RM sintered pad; (**b**) secondary electrons SEM micrograph of the same sample.

**Figure 2:**
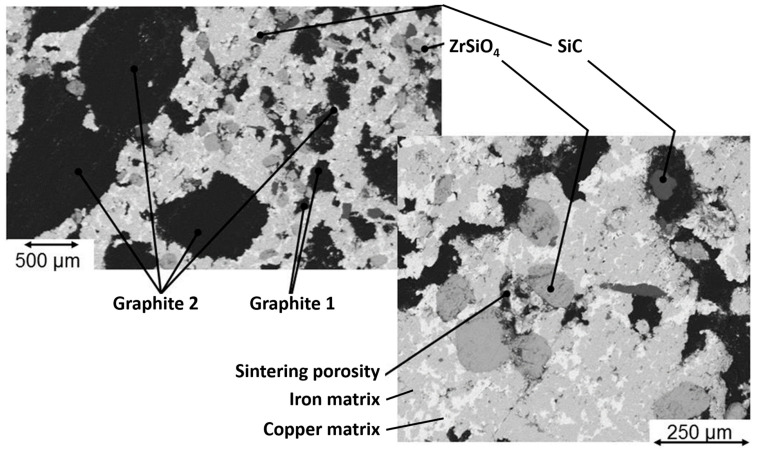
Back-scattered electrons SEM micrographs of the RM pad.

The sintering process implies several modifications of the constitution of the global material. Tin is detected in the copper phase and manganese in the iron phase. The high temperature reached during the sintering process also allows the diffusion of carbon into iron. This is revealed via both optical (Figure 1a) and electronic (Figure 1b) microscopy, as a fair amount of pearlite can be observed in the iron matrix, and locally even a hypereutectoid-characteristic cementite grain boundary between pearlite grains might be found. A further SEM analysis (Figure 2), along with EDS analyses of the RM pad, showed the presence of the following:

- A metallic matrix composed of intertwined tin-enriched copper and of manganese-enriched perlite and ferrite. The presence of distinct ferrite grains and of hypereutectoid-like pearlite grains separated by cementite boundaries proves that most of the carbon has been introduced in iron via diffusion during the sintering process. According to the literature, this carbon might come from SiC if the temperature is above 1000 °C [33], which was not the case. The most obvious source of carbon is graphite particles.
- Both ceramics (SiC and ZrSiO_4_) are seemingly (and unsurprisingly) unaltered, both in size and shape.
- The two different graphite types can still be identified, with small grains of “graphite 1” and big porous grains of “graphite 2”.

**Figure 3:**
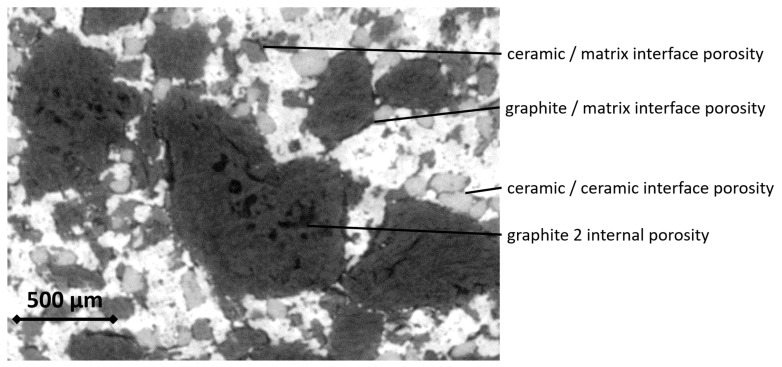
Tomographic micrograph of the RM pad. Contrast reflects density. From darkest to lightest: porosities, graphite, SiC and ZrSiO_4_, ferrite and cementite, copper.

The sintered RM pad shows several characteristic defects that are shown in Figure 3. Porosities are a normal defect in sintered materials, but other, more specific defects appear. The metallic matrix seems to be strongly bounded, as no open interfaces can be detected on the SEM images (Figure 2). Conversely, graphite particles are weakly bounded to the matrix, with clear discontinuities that appear at the interface with the matrix in tomography (Figure 3), but also on SEM micrographs as the interface between graphite and metal is irregular. Emerging graphite particles appear to be slightly under the plane surface, which is consistent with an absence of chemical bounding between the two phases.

In some cases, the metallic matrix did not “wet” completely the ceramic or graphite grains; this resulted in other inter-particles discontinuities. Finally, although this is not a defect coming from the process, it is important to bear in mind that Graphite 2 grains are porous. The graphitization process induces very high temperatures (usually >3000 °C) to provoke the evaporation of all the impurities and the crystallization of graphite, which results in the existence of large porous zones inside the particles (Figure 3).

### 3.2. Compressive Tests

**Figure 4:**
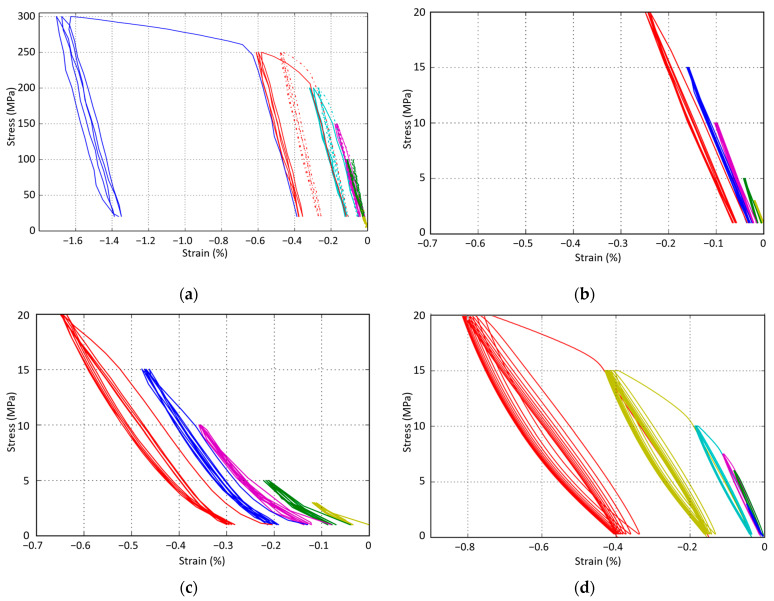
Stress–strain results for progressive, cyclic (5 cycles/load, 1 colour per load) compression tests: (**a**) MM (full) and MMCs (dotted lines); (**b**) MMCsG1; (**c**) MMCsG2; (**d**) RM materials.

The stress–strain curves obtained from the compressive tests show two main results: the elastic modulus and yield strength are strongly affected by the presence of 20% wt. of graphite (Table 3); and materials containing the big, porous particles of Graphite 2 show a hysteresis phenomenon between loading and unloading (Figure 4).

The elastic modulus of the samples was measured using the second loading at each load step. The yield strength was estimated graphically. As expected, the MM and MMCs samples show ten-times-higher strength and rigidity than the MMCsG1 and MMCsG2 samples.

Nonetheless, the MM sample has considerably lower mechanical properties than iron (E = 200 GPa) or copper (E = 130 GPa). A lower modulus can be attributed to the development of cracks through the structure, but a part of it can also be attributed to an overestimation of the actual section due to porosities.

The very low modulus values measured for MMCsG1 and MMCsG2 samples show the controlling influence of graphite particles. The modulus of the RM sample is higher than that of MMCsG2 and lower than that of MMCsG1. This shows that the mechanical behaviour of the samples under compressive stress is greatly influenced by the behaviour of its graphite particles, as they are the most deformable particles and represent a fair percentage of the volume. Furthermore, Graphite 2 seems to imply less rigidity, which can be expected as it is more porous than Graphite 1.

**Figure 5:**
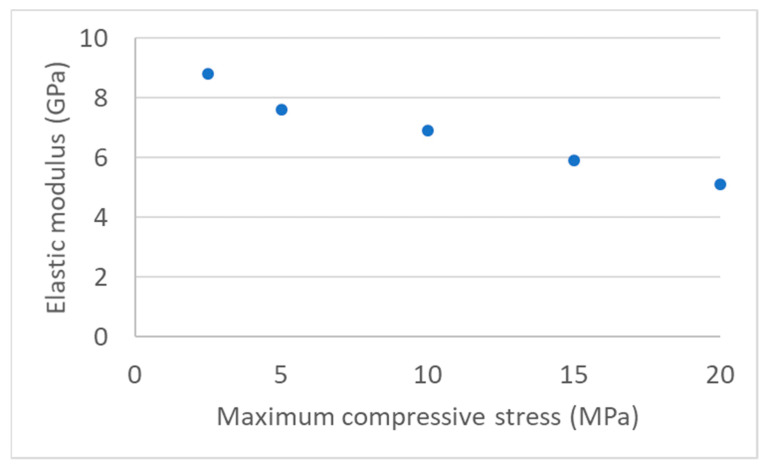
Evolution of the RM elastic modulus measured during the second loading at each new maximum load step.

**Figure 6:**
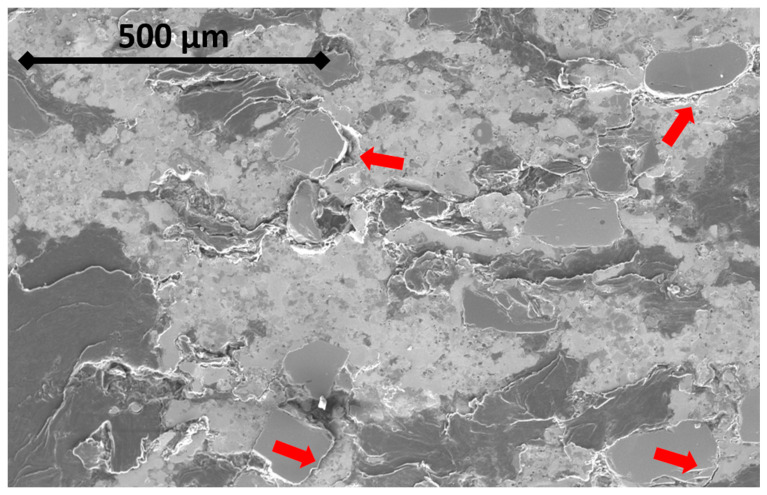
Secondary electrons SEM micrograph of the RM pad after the compression test. Red arrows point at some unbound ceramic/matrix interfaces.

Figure 5 shows the evolution of the RM modulus according to the maximum load during the compressive test. No significant change could be measured between cycles operated at the same maximum load, whereas the elastic modulus regularly drops when a new maximum load is reached. The micrograph shown in Figure 6 confirms that the drop in the modulus can be attributed to damage, as cracks extend at interfaces between matrix and particles and develop through the matrix. 

Finally, a hysteresis phenomenon is observed for RM and MMCsG2 samples, but not in the other cases. As soon as a plastic deformation is reached, the unloading step of the cycle can be separated into a first “high-modulus” unloading down to 3–5 MPa, followed by a “low-modulus” unloading down to 1 MPa. The following loading stage seems to be more linear. The amplitude of the hysteresis cycle grows with the load. 

**Figure 7:**
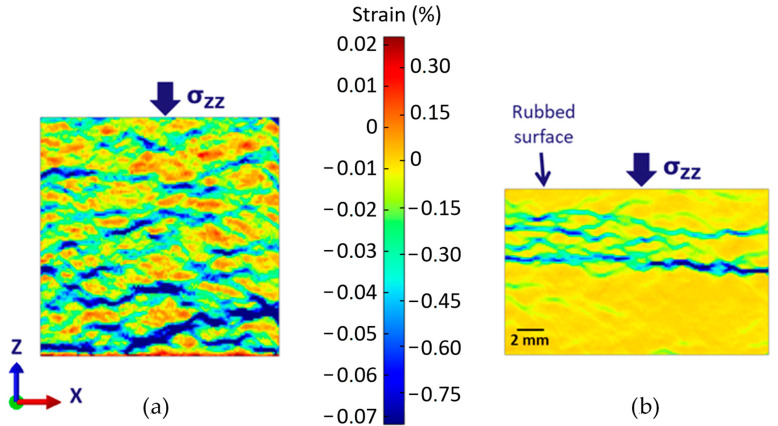
DIC analysis of the RM pad, compressed at 20 MPa maximum load, (**a**) before and (**b**) after the braking test.

As this hysteresis phenomenon only concerns samples containing Graphite 2, it is tempting to attribute most of the compressive load accommodation to Graphite 2 particles. Figure 7 shows the DIC strain fields of a 1–20 MPa elastic loading of the RM sample before and after the braking test. Both colour scales have the same amplitude. DIC image treatment shows that the most displaced areas on the front surface correspond to large Graphite 2 particles and to interlinking areas. The limitation of this analysis is obviously the lack of 3D information. Nonetheless, the micrograph (Figure 6) shows that between graphite grains, cracks develop through the matrix along ceramic particles.

At this stage, several conclusions can already be drawn. The overall mechanical behaviour of the RM pad in compressive tests strongly depends on the contribution of its weaker particles: graphite. Moreover, the elastic modulus of the RM pad (8.8 GPa) is halfway between that of the slightly more rigid MMCsG1 (10.9 GPa) and that of MMCsG2 (5.2 GPa), reflecting the mixed composition of RM (7% wt. of graphite 1 and 13% wt. of graphite 2). 

To underline the major role played by graphite in the mechanical behaviour of the pad, it is important to remember that the 20% wt. of graphite occupy a large volume in the pad. Graphite in general, and our porous graphite 2 in particular, has a low density. Although it is difficult to estimate a meaningful value of graphite density in the sintered material, we can estimate that graphite occupies about 40 to 50% of the volume of the sample. 

We suggest that the factor 10 drop in the elasticity modulus between strong graphite-free and weak graphite-full pads indicates that the amount of graphite is sufficient to accommodate a considerably higher displacement than what the metallic matrix allows, with or without ceramics. Within the graphite-full samples, MMCsG2, with its big, porous graphite 2 particles, induces the hysteresis behaviour that is observed for both MMCsG2 and RM pads. This might be a consequence of the massive porosity of the graphite particles, which could accommodate a huge displacement for virtually no load, before a more classical elastic behaviour can be set. Now, this does not explain why loading and unloading are not identical. This two-phenomena approach (porosity and elasticity) can explain the hysteresis if it allows us to differentiate loading and unloading behaviours. During the loading, porosities can absorb the displacement at the very beginning, then the elastic behaviour of graphite takes place. But the two phenomena continue to coexist. If the yield strength is reached, plastic deformation can occur in graphite, especially in graphite 2, since it is highly crystalline. The unloading steps show a higher slope than the loading steps, at least in the first stage of unloading. Our interpretation is that the elastic behaviour is not delayed, while the porosities can only reshape either when the majority of the load has been cancelled, or simply at a slower pace. Moreover, graphite 2 grains are considerably larger that graphite 1 grains, allowing a larger strain.

Although determining the yield strength is not precise in our conditions, it appears that graphite-full samples show a very low yield strength, always under 20 MPa, whereas graphite-free samples reach about 120 MPa. The difference is massive, and it seems to indicate that the metallic matrix will not be affected by the 20 MPa compressive tests. Even a hypothetic lattice structure, made exclusively with the metallic matrix and voids, should sustain 60 MPa. So, this yield strength under 20 MPa shows that graphite grains strain is predominant to accommodate the stress.

To go further, we need to remember that crystalline graphite is made of strong graphene sheets linked together by weak chemical bonds. This means that the border between elastic and plastic behaviour is weaker than for metals. A good part of the displacement can be accommodated through relative movement of graphene sheets, which would correspond to the definition of plasticity, but this weakness in the bonds also means that a heterogeneous microstructure could induce a back-displacement of the graphene sheets during unloading. 

Nonetheless, graphite particles do not make a homogeneous layer. They are scattered in the matrix. If we could integrate the graphite amount in every position of the (x,y) plane, or simply mentally pile up the components, we would probably conclude that graphite represents a quite steady part of the sample, whatever the (x,y) position. This is precisely the meaning of the representative elementary volume hypothesis that we made earlier to justify the volume of the compressive test sample. The advantage and the disadvantage of this hypothesis is that it hides the fundamental heterogeneity of the sample. 

Now, if we rethink our sample, keeping in mind the heterogeneity, this brings a new question: what happens to the metallic matrix as the graphite particles accommodate displacement? In other words, what happens in the regions where graphite is either over or under-represented? 

The increasing load applied for each series of compressive cycles helps us answer these questions. Figure 5 shows the evolution of the elastic modulus for the RM sample as a function of the maximum load during the second cycle of each series. As the modulus drops, it reaches values closer and closer to that of MMCsG2. There are two complementary explanations: damage, that is to say, the development of cracks in the matrix, and consequently the preponderance of Graphite 2 particles in the global behaviour. 

As Figure 6 shows, cracks develop throughout the matrix, either at interfaces between matrix and ceramic particles, through sintering porosities, or sometimes between metallic grains of the matrix. This means damage occurs in the matrix at very low loads. The heterogeneity of the microstructure implies that the displacement cannot be accommodated everywhere at the same level by graphite only. And if the interface between graphite and metal is fragilized by the difference in the capacity of both phases to accommodate displacement, it is also true at the interface between ceramics and metals. As we do observe cracks developing through the matrix and interfaces, this can only mean that the actual local load is sufficient to allow damage, and thus a drop in the elastic modulus. 

### 3.3. Structure of the Pad after the Braking Test Series

High-energy braking tests were performed with sintered RM pads. These series implied relatively low load as the global pad/disc contact surface is quite large. The maximum stress that was reached is 0.758 MPa. Nonetheless, considerable damage is observed on the pad. Local stress is probably much higher, and the amount of dissipated energy during braking makes the temperature rise considerably. Thermocouples positioned at 3 mm depth reached 950 °C, and 810 °C at 6 mm depth. Furthermore, hot spots were observed on the disc surface during the braking test. 

**Figure 8:**
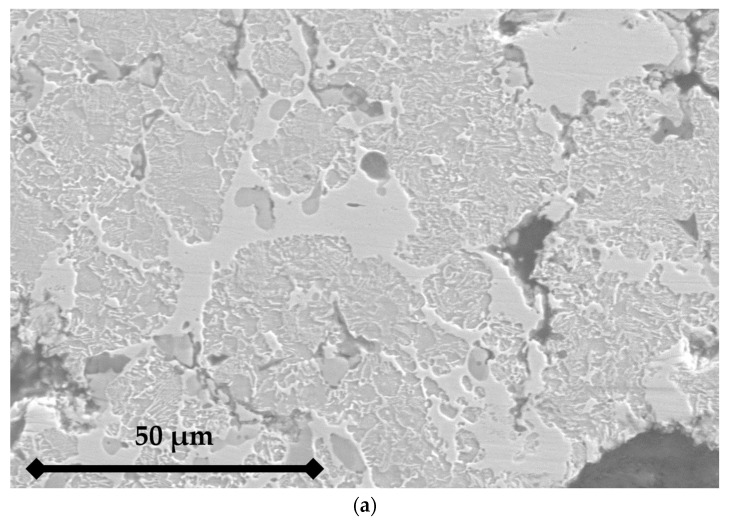

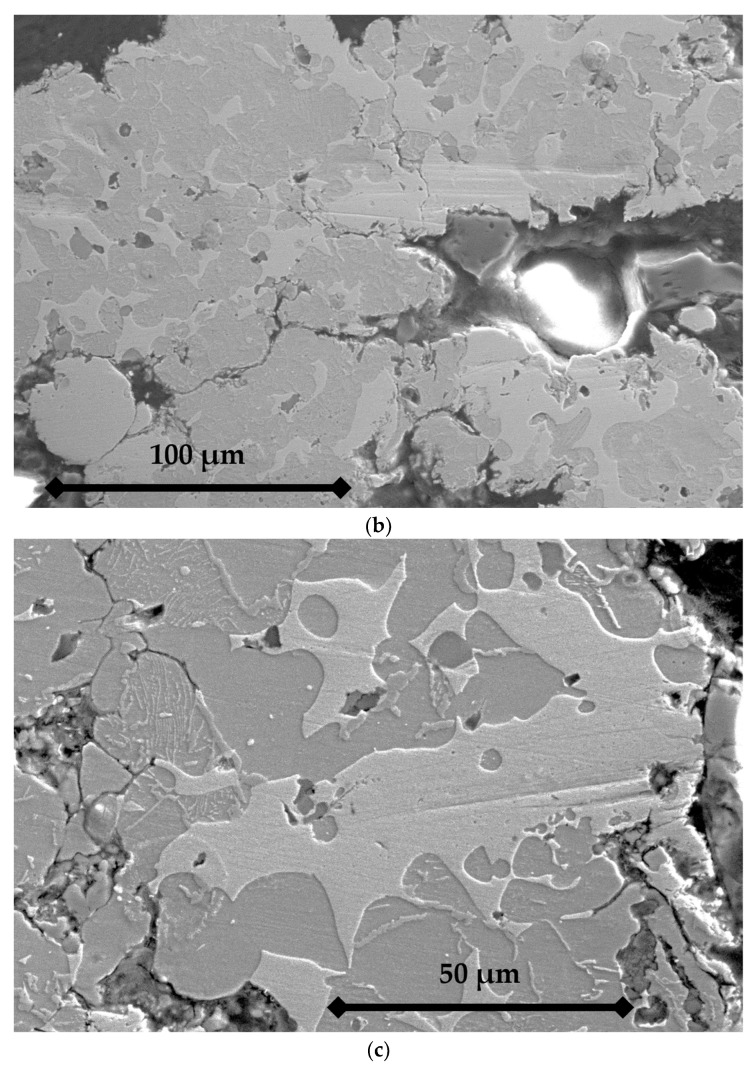
Secondary electrons SEM micrographs of the RM pad after the braking test: (**a**) top 2 mm layer; (**b**) intermediate (2–15 mm) layer; (**c**) bottom layer.

After the braking test, three layers can be distinguished in the pad (Figure 7) according to the DIC-measured strain. The height difference between the original and the worn sample is due to wear during the braking test. The bottom half has been less affected by the braking test than the rest: it shows a very homogeneous strain at this scale that corresponds to the behaviour of the materials prior to the braking test. Figure 8c is a SEM micrograph that shows this bottom layer has a very similar microstructure to the virgin RM sample. All the usual constituents are found. Some cracks are visible. The one at the right of the micrograph develop through a network of particles boundaries and pre-existing porosities that are common with the sintering process. The crack on the left of the micrograph also links pre-existing porosities and particle boundaries, but it is also clear that cracks develop at the interface between ferrite and pearlite in the metallic matrix. This can explain a good part of the difficulties met by companies seeking to produce copperless pads. The carbon diffusion phenomenon may weaken the interfaces between ferrite and pearlite. 

Conversely, the interfaces between copper and iron do not seem to lead to any cracking. This means that the sintering process works quite well to link copper and iron (either ferrite or pearlite), although our sintering temperature is 150 °C under the industrial iron–copper liquid-phase sintering process temperature [34]. The fact that we observe ferrite and perlite means that the sintering process allows the allotropic transformation of ferrite into austenite. Carbon diffusion into austenite that happens during sintering results during a slow cooling in the ferrite/pearlite microstructure. Now, how does this make weaker interfaces between ferrite and pearlite than between iron and copper? 

First, a close observation of the micrograph shows that micrometric and submicrometric particles can be found in the porosities and in the path chosen by the cracks. This might be another consequence of the sintering process. First, the powders granulometry are known, but without guarantee that the 100 µm particles have not gathered smaller particles on their surfaces. For example, Hessels et al. observe, in reductive conditions, the formations of nanostructured whiskers on the surface of reducing iron oxide/iron grains [35]. Second, the first step of the process is a mixing operation that aims at making a homogeneous mix before sintering. This stage might help scatter some of these submicrometric particles in the mix. Sintering would then allow a good adhesion between metals, but these small, inert particles can either gather locally in future porosities of the pad (that is to say, in places the metallic matrix particles could not reach) or simply at the interfaces between metallic grains, making theses interfaces weaker than what was expected. 

Secondly, it is useful to keep in mind that the sintering process is adapted to copper, not to iron. The maximum temperature is 950 °C, which is far from the liquidus curve of the iron–carbon diagram, and far from the industrial temperature of sintering for pure iron at 1120 °C, or, as an example, for iron–nickel alloys at 1145 °C [34]. In the industry, iron–copper sintering is carried out at a temperature superior to the melting temperature of copper in order to allow a liquid copper phase to wet the iron solid skeleton. Diffusion phenomena are considered too slow to take place even at these temperatures, especially if the copper phase does not melt at any stage [34]. Nonetheless, the presence of tin in the copper phase lowers the liquidus temperature. As a consequence, interfaces between copper grains are sound. Interfaces between copper and iron phases are mostly sound, as much as copper has managed to wet the iron grains. Conversely, interfaces between two iron particles cannot be strongly bonded if no copper is involved. Consequently, if most of the pearlite/ferrite interfaces result from a classic diffusion process during the cooling of austenite, others come from a defect of the sintering process between different particles of iron. This explanation is consistent with the presence of the small ceramic and graphite particles inside the cracks that separate pearlite and ferrite on Figure 8b,c. Another consistent consequence is the compressive behaviour of the pads: as previously underlined, the yield strength of the RM sample is considerably lower that of the MM and MMC samples.

Getting back to compressive tests after sintering, but before emergency brakes tests, the compression elastic modulus of the MM and MMC samples were lower than those of iron or even copper. This is also consistent with an insufficient sintering process for iron. 

**Figure 9:**
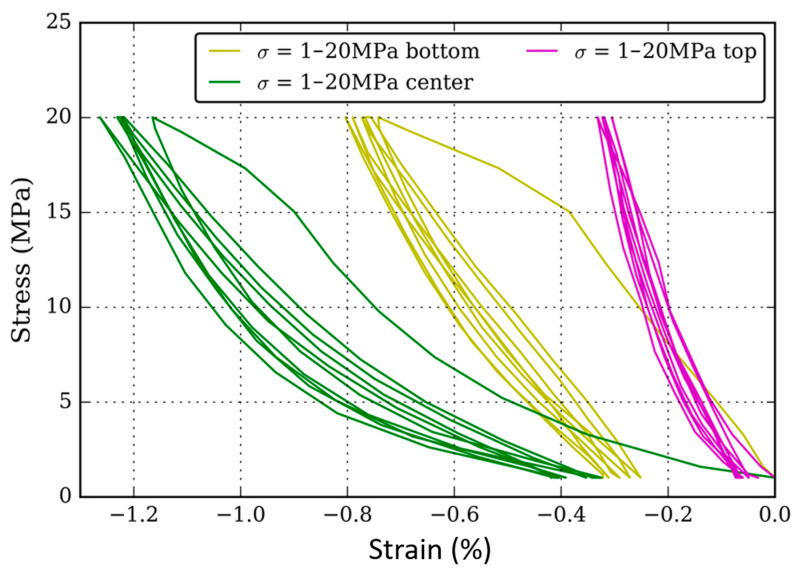
Stress–strain compression tests for five 1–20 MPa cycles applied on different layers of the worn pad: (green) intermediate (2–15 mm depth) region; (gold) bottom region; (pink) top layer (first 2 mm).

The compression test (Figure 9) on this bottom area is quite consistent with what was observed before the braking test: the elastic modulus measured for the 20 MPa maximum load series is about 4 GPa, corresponding to a damaged RM material. 

The intermediate zone is crossed by several major cracks (Figure 8b) that deeply affect its mechanical behaviour (Figure 9). This confirms the important role played by voids in the structure: the low modulus and its progressive drop with the increasing load. 

In this area, even if the temperature was high enough to allow once again the reformation of austenite, it was not high enough to provoke the melting of copper. As a consequence, at best, the same mechanisms described for the sintering process apply: a slow enough cooling of austenite provokes the formation of ferrite or cementite (according to the local carbon percentage), then of pearlite. These transformations do not affect the original problem: bonds between iron particles are still far from an industrially valid sintering. 

Furthermore, either in the bottom or intermediate zones, the hysteresis phenomenon is still observed, but with less intensity. This might be explained by the fact that damage has created more void in the samples, allowing more accommodation that do not need to be carried by graphite 2 anymore. 

Finally, the top layer, reaching down to 1.5 mm in depth, shows very different microstructure (Figure 8a) and mechanical behaviour (Figure 9) from the rest. The two observations are linked. The microstructure shows that the matrix has been chemically and physically modified. Pearlite is the main constituent, whereas next to no ferrite could be found. This can only be explained by diffusion of carbon from graphite into iron. This phenomenon must create voids at the interface between the two phases. Nonetheless, this may not affect the mechanical properties because the original interface between iron and graphite was not mechanically consistent. 

For this diffusion process to happen at a measurable scale, the temperature has to reach the eutectoid temperature, at 727 °C, a temperature that was easily reached at 3 mm from the surface according to the thermocouple. We can assume from the measured temperatures that iron has been fully converted to austenite in the top layer. The microstructure also indicates that cooling was fast enough to prevent the formation of bainite, but not slow enough to ensure the formation of an equilibrium pearlite. Finally, the microstructure also reveals that many small porosities are found within the metallic matrix, usually around ceramic particles. This is reminiscent of the solidification process in which copper would solidify, occupying less volume than the liquid. In this case, the localisation of porosities at the ceramic/copper interface can be explained by three factors. First, the ceramic particles would not easily bond to metals. Second the thermal exchanges between copper and ceramics are slower than between copper and the rest of the pad (iron and graphite), so the ceramic particles will slow down cooling, making copper solidify later at their contact, thus provoking the porosity. And third, this does not happen at the iron/ceramic interfaces because pearlite will start melting at about 1300 °C, a temperature that is not reached during the test.

**Figure 10:**
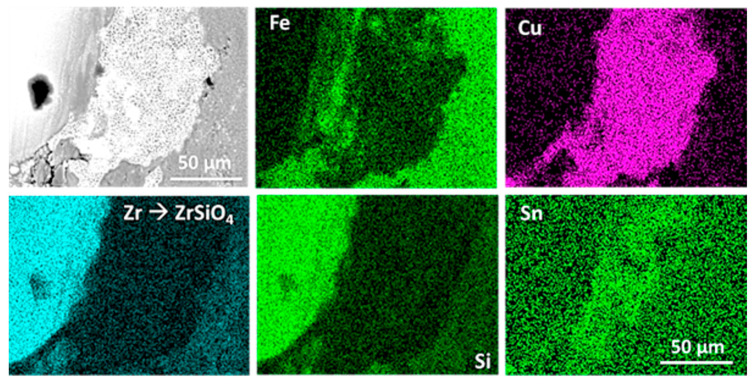
Secondary electron SEM micrograph with X-ray maps of a top-layer area of the RM pad after the braking test showing the re-solidification of copper and the presence of small ZrSiO_4_ particles.

Figure 10 is a SEM micrograph of the top surface of the RM pad after braking, with an X-ray map of Fe, Cu, Zr, Si, and Sn. This micrograph shows that sintering affects the composition of the introduced elements. For example, tin is solved into copper, making the melting temperature diminish. The interface between Cu and Fe does not show discontinuities. Small ceramic particles at the bottom left of the micrograph disturb the quality of the sintering: not only are they unaffected by the sintering process, but they also prevent the metallic matrix from reaching down this scale of interfaces. 

Figure 10 also shows that the microstructure of the sintered pad evolves with high-intensity stress. Copper has melted and re-solidified. This can explain the re-increase in rigidity of this part of the sample (Figure 9). The elastic modulus has risen to values comprised between 9 to 12 GPa, which cannot be explained by damage. It seems that the melting and solidification of copper has enhanced the mechanical properties by healing cracks and bonding with iron [34].

## 4. Discussion

These observations lead us to the following scenario to explain the specific failure of sintered iron/copper matrix pads exposed to very intense braking stress:

- The mixing stage before the sintering process might disseminate unwanted submicronic particles among the metallic particles that will form the matrix. This induces weaker interfaces between metallic grains, especially between ferrite and pearlite.
- The sintering stage induces carbon diffusion from the graphite particles into iron: pearlite is formed in the matrix. The complex composition of the metallic matrix suggests the setting of unusually low sintering temperature that allows good sintering between copper particles but leaves most of the interfaces between iron-rich particles unsintered.
- The sintering process also induces the presence of porosities. These porosities seem to gather around groups of small ceramic particles, smaller than what was supposedly introduced.
- The resulting pads show a very weak compressive behaviour, with an elastic modulus of about 10 GPa and yield strength under 15 MPa, whereas graphite-free samples made in the same conditions showed an elastic modulus of 100 GPa and a yield strength of about 120 MPa. This proves the paramount importance of graphite in the accommodation of displacement.
- Among graphite particles, Graphite 2 (bigger, more porous and crystalline) shows a preponderant role in the mechanical behaviour as it lowers the mechanical properties and induces a hysteresis phenomenon that we explain by the role of its internal porosities.
- The heterogeneity of the pad induces strong variations of local load and displacement that result in the opening of cracks at interfaces between the metallic matrix and ceramic particles (if they were bound together in the first place).
- Discontinuities at the interface between ferrite and pearlite appear to take their origin in the sintering process rather than in the annealing induced by the braking test.
- This ferrite–pearlite interface may be weakened by the presence of the aforesaid small ceramic particles and by possible voids allowed by the diffusion of carbon from graphite into iron.
- High-energy braking allows a thick part of the pad to reach very high temperatures, with heavy consequences on the microstructure and mechanical properties. Thermocouples and physical phenomena such as carburizing of iron and copper melting show that temperatures of 1000 °C have been reached.
- The gradient of temperature induces a variety of mechanical behaviours. At more than 10 mm from the surface, the microstructure and compressive mechanical properties seem unaffected.
- An intermediate zone is strongly affected, massive cracks develop throughout the sample, in the normal plane from the load.
- The top layer, reaching very high temperatures, is strongly modified. The diffusion of carbon into iron, started during the sintering process at a moderate 950 °C, follows on. Copper melts and resolidifies, healing the cracks in the process, which results in slightly enhanced elastic modulus and yield strength.
- Finally, this carburizing phenomenon that happens during the sintering process and the braking test might explain why companies have been unable to produce satisfying copperless pads, as it implies a weakness of interface within the metallic matrix, at the pearlite–ferrite interfaces.

## 5. Conclusions

Failure of metallic-matrix braking pads in high-energy, emergency-type rail braking has been linked with defects in the microstructure, with their origin lying in the sintering process.

Introducing major amounts of iron within the copper matrix does not allow for an optimized set of parameters during sintering. A higher temperature would allow a better sintering of copper and iron, but it would also imply more diffusion of carbon into iron phases, leading to weaker interfaces. A lower temperature would prevent copper from sintering.

The consequences are low mechanical properties in compression that are dominated by the behaviour of the largest particles of graphite.

The high-energy braking test allowed the surface of the pad to reach the melting temperature of copper, which resulted in a gradient of mechanical behaviours: the top layer was stronger owing to cracks healing, whereas the subsurface was much damaged as the braking test resulted in an over-sintering that revealed the carburizing and interface weakening mechanisms.

## Figures and Tables

**Table 1:** Size distribution of the main components of the RM pads.

	Iron	Copper	SiC	ZrSiO_4_	Graphite 1	Graphite 2
Size (µm)	<220	<100	20–260	80–320	50–500	300–1250

**Table 2:** Formulation of the pads, in wt%.

	Iron	Copper	SiC	ZrSiO_4_	Graphite 1	Graphite 2	Fillers
RM	34	26	2	8	7	13	10
MM	49	37	-	-	-	-	14
MMCs	43	32	3	10	-	-	12
MMCsG1	34	26	2	8	20	-	10
MMCsG2	34	26	2	8	-	20	10

**Table 3:** Elastic modulus and yield strength of the virgin pads in compressive tests.

	E (GPa)	Re (MPa)
MM	96	120
MMCs	102	120
MMCsG1	10.9	16
MMCsG2	5.2	10
RM	8.8	8

## Data Availability

Data can be accessed on demand to the main authors.
